# Method Standardization for Conducting Innate Color Preference Studies in Different Zebrafish Strains

**DOI:** 10.3390/biomedicines8080271

**Published:** 2020-08-03

**Authors:** Petrus Siregar, Stevhen Juniardi, Gilbert Audira, Yu-Heng Lai, Jong-Chin Huang, Kelvin H.-C. Chen, Jung-Ren Chen, Chung-Der Hsiao

**Affiliations:** 1 Department of Bioscience Technology, Chung Yuan Christian University, Chung-Li 320314, Taiwan; siregar.petrus27@gmail.com (P.S.); stvn.jun@gmail.com (S.J.); gilbertaudira@yahoo.com (G.A.); 2 Department of Chemistry, Chung Yuan Christian University, Chung-Li 320314, Taiwan; 3 Department of Chemistry, Chinese Culture University, Taipei 11114, Taiwan; lyh21@ulive.pccu.edu.tw; 4 Department of Biological Science & Technology, College of Medicine, I-Shou University, Kaohsiung 82445, Taiwan; hjc@mail.nptu.edu.tw; 5 Department of Applied Chemistry, National Pingtung University, Pingtung 900391, Taiwan; 6 Center for Nanotechnology, Chung Yuan Christian University, Chung-Li 320314, Taiwan

**Keywords:** zebrafish behavior, color preferences, toxicity assessment

## Abstract

The zebrafish has a tetrachromatic vision that is able to distinguish ultraviolet (UV) and visible wavelengths. Recently, zebrafish color preferences have gained much attention because of the easy setup of the instrument and its usefulness to screen behavior-linked stimuli. However, several published papers dealing with zebrafish color preferences have contradicting results that underscore the importance of method standardization in this field. Different laboratories may report different results because of variations in light source, color intensity, and other parameters such as age, gender, container size, and strain of fish. In this study, we aim to standardize the color preference test in zebrafish by measuring light source position, light intensity, gender, age, animal size to space ratio, and animal strain. Our results showed that color preferences for zebrafish are affected by light position, age, strain, and social interaction of the fish, but not affected by fish gender. We validated that ethanol can significantly induce color preference alteration in zebrafish which may be related to anxiety and depression. We also explored the potential use of the optimized method to examine color preference ranking and index differences in various zebrafish strains and species, such as the tiger barb and glass catfish. In conclusion, zebrafish color preference screening is a powerful tool for high-throughput neuropharmacological applications and the standardized protocol established in this study provides a useful reference for the zebrafish research community.

## 1. Introduction

Color perception, an important trait that allows animals to recognize food, predators, shoaling, mating choices, and hiding places, has been reported to influence the learning behavior and memory formation of zebrafish [1,2]. Recent studies showed that zebrafish behavior could be used to evaluate the neurotoxicity of drugs [3,4]. Several transgenic zebrafish models [5,6] have been developed to assess behaviors based on various color cues and visual stimuli, such as T-maze [3,7], passive avoidance [8,9], Y-maze [10] and cross maze [6]. The pathway between the photoreceptor and the color spectrum recognition in animals and lower vertebrates are evolutionarily conserved [6]. Zebrafish have four different cones to distinguish UV and visible wavelengths. Specifically, the UV-cone has a peak sensitivity at 362 nm, the S-cone has a peak sensitivity at 417 nm, the M-cone peak sensitivity is at 480 nm, and the L-cone has a peak sensitivity at 556 nm [11]. Zebrafish larva responds to light at 3.5 day-post-fertilization (dpf), displays mobility from 5dpf onward, and is able to discern color from the early age of 5 dpf [6].

Several color preferences have been commonly demonstrated in zebrafish; however, the results were sometimes contradictory (summarized in Table A3). For example, either red [11,12] or blue [1,3,6,7,13,14] has been reported with the highest color preference ranking in zebrafish. Different color preference ranking results reported by different laboratories may be due to variations in the light source position (provided from the top or bottom positions), color intensity (different illumination lux), and/or physical parameters such as fish age and gender [1,2,3,6,7,11,13,14]. The variability of the assays can be overcome by standardizing the experimental conditions to ensure consistent results and ease in interpretation. In this study, we aim to standardize the innate color preference test condition in adult zebrafish by evaluating the potential effect of light source position and intensity, social interaction, gender, age, and strain of the tested fish. Furthermore, we applied the optimized protocol to evaluate the color preference difference in adult zebrafish exposed to ethanol to explore the potential uses of this setting for toxicity assessment.

## 2. Material and Methods

### 2.1. Animal Ethics and Animal Used in the Study

All the experimental protocols and procedures involving zebrafish were approved by the Committee for Animal Experimentation of the Chung Yuan Christian University (Number: CYCU104024, issued date 21 December 2015). All experiments were performed in accordance with the guidelines for laboratory animals. For intraspecies comparison, six zebrafish strains, AB, Tübingen long fin (TL), Wild Indian Karyotype (WIK), golden, absolute, and pet store-purchased (PET) were used. The TL zebrafish phenotype shows longer fins compared to the AB strain. The TL zebrafish are homozygous mutants for leo^t1^ and lof^dt1^. leo^t1^ is a recessive mutation that causes spotting in adult zebrafish. The other mutation lof^dt1^ is a dominant homozygous viable mutation causing long fins [15,16]. The absolute mutant fish is a double mutant fish carrying the *ednrb1*^b140^ and *mitfa*^b692^. In zebrafish, *ednrb1* is important in adult pigment pattern formation [17,18]. This fish is also carrying the mitfa^b692^ mutation [19] which renders the skin transparent. The absolute mutant lacks melanophore, xanthophore, and most of the iridophore cells causing the transparent phenotype [20]. The golden zebrafish exhibited lightening of the pigmented stripes and golden phenotype. The golden phenotype is characterized by the mutation in *slc24a5*. This mutation causes a delayed and reduced development of melanin pigmentation in zebrafish. The mutation produced a lighter stripes in golden zebrafish [21]. The WIK strain was originally derived from a wild-caught line and is highly polymorphic compared to the AB strain. They are often used for genome mapping in zebrafish because of their characteristic of being highly polymorphic [22]. Meanwhile, the PET zebrafish were derived from a local aquarium in Taiwan, they represent the wild-type genetically and presumably have a heterogeneous genetic background [22]. For interspecies comparison, another two teleost species of tiger barb (*Puntigrus tetrazona*) and glass catfish (*Kryptopterus vitreolus*) were used. Four zebrafish strains, AB, TL, WIK, and absolute, were obtained from Taiwan Zebrafish Center at Academia Sinica (TZCAS), and the PET and the golden zebrafish strains, as well as the tiger barb and glass catfish, were purchased from a local pet store.

### 2.2. Color Preference Assay

**Figure 1:**
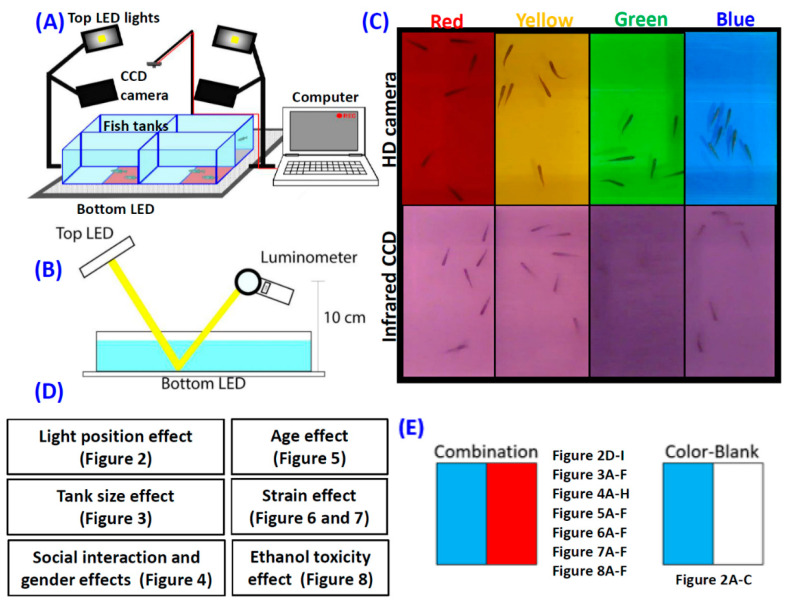
Experimental setup for color preference assay in zebrafish. (**A**) Schematic of the experimental setup and a picture of the experimental setup used for measuring zebrafish color preference in this study. (**B**) Schematic showing the position of the luminometer to measure the top and bottom light intensity. (**C**) Comparison of the images collected from regular Couple-charged device (CCD) (top) and infrared CCD cameras (bottom). (**D**) Experimental design and specific aims of this study. (**E**) Schematic illustrating tank area with color plate combination and color blank design.

**Figure A1:**
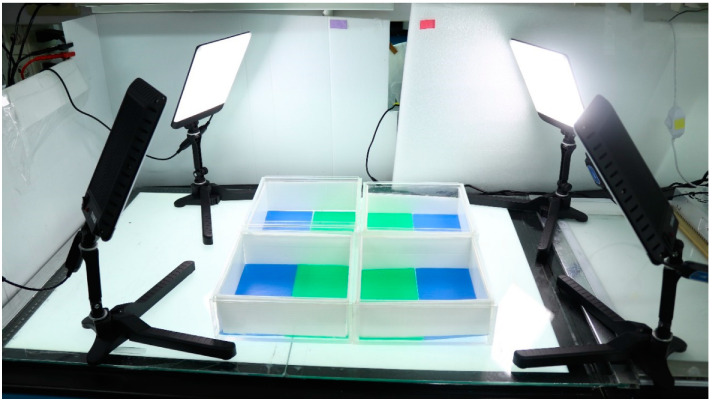
Instrument setting used to perform the color preference test in zebrafish.

The assay was conducted in a 21 × 21 × 10 cm acrylic tank and filled with 1.5 L of filtered water. Each half of the tank area with a size of 10.5 × 10.5 was covered with a color plate combination with lux intensity as listed in Table A1. All the color preference assays conducted in this study took place in the room temperature condition, which is around 26–28 °C. The temperature condition is the same with zebrafish maintenance conditions. Four 30W LED lights were positioned above (SerRickDon, Shenzhen, China) and one 60 × 60 cm 24W LED plate (Lumibox, Shenzhen, China) was positioned below the fish tanks to provide different combinations of illumination positions and intensities (Figure 1A and Figure A1). A high-quality Couple-charged device (CCD) camera with a maximum resolution at 3264 × 2448 pixels and 30 frames per second (fps) frame rate (ONTOP, M2 module, Shenzhen, China) and an infrared (IR) camera (700–1000 nm detection window) with a maximum resolution at 1920 × 1080 pixels and 30 fps frame rate (3206_1080P module, Shenzhen, China) was used to record the fish locomotion activity. We also measured the reflectance of all color plates (Figure 1B and Table A2).

The high-quality CCD camera was used to record the blue–green color plate combination since the IR camera was not feasible in the tank with a blue–green color combination. The other color combinations (red–yellow, red–blue, red–green, yellow–blue, and yellow–green) were recorded by using the IR camera to increase the video signal-to-noise ratio (Figure 1C). The light intensity was measured by using a luminometer (Peakmeter Instruments Co., Ltd., Shenzhen, China). The luminometer’s sensor was positioned facing the tank 5 cm above the water level to measure the light intensity (Figure 1B). The arrangement of four experimental tanks is shown in Figure 1A and Figure A1 to increase the recording output. The wild-type AB strain zebrafish was used in this experiment. The zebrafish age used in this study is around 5–6 months old. Four experimental tanks were arranged side by side into a 2 × 2 array as shown in Figure 1A and Figure A1 to increase the recording output. The experiments were divided into several different categories such as light position, tank size, social interaction, age, and gender to compare different variables that may affect the color preference result (summarized in Figure 1D). The light position, tank size, social interaction, and age were conducted in the color plate combination experiments. Social interaction and gender experiments were also conducted with half of the tank covered in color plate and the other half not covered (blank) (Figure 1E). All the experiments were conducted with a total of 24 fishes used in each category, except in ethanol-treated fish with a total of 12 fishes used in each ethanol-treated category.

The tank size comparison experiment was conducted to compare a 20 × 20 × 10 tank filled with 1.5 L and a 30 × 30 × 10 tank filled with 4.2 L which have the same height: the water-filled ratio. For comparing the effect of social interaction towards color preferences, the swimming activity of single zebrafish in one tank was compared with six zebrafishes shoaling in one tank. The gender effect was assessed by measuring the 5-month-old male and female zebrafish color preferences separately. The zebrafish age used for age-dependent color preference study was 3, 5, and 12 months old with a total of 24 fishes used in each age group. For all the color preference tests, the zebrafishes were placed into the water tank and immediately performed video recording by using IR or conventional camera for 30 min. No habituation time was performed in the experimental tank, and zebrafish movement was analyzed for 30 min in this study. Total Recorder software (High Criteria Inc., Richmond Hill, ON, Canada) was used to capture the video at a resolution of 1920 × 1080 pixel using the IR or conventional camera.

### 2.3. Data Analysis

The fish locomotion within 30 min of the recorded video was analyzed by using an open source idTracker software (Ver. 2.1, Cajal Institute, Madrid, Spain) [23]. The XY coordinates obtained by idTracker software was used to calculate the appearing times in different partitions of the zebrafish by using Microsoft Excel. The choice index equation was used to calculate the preferences of the zebrafish color preferences [24,25].

(1) $$\begin{array}{c} Choice\;index=\\ \;\frac{Time\;stay\;in\;color\;partition\;\left(s\right)-Time\;stay\;in\;a\;second\;color\;partition\;\left(s\right)}{Total\;video\;time\;\left(s\right)} \end{array}$$

### 2.4. Statistics

One-way ANOVA was used to analyze single group data for combined time point results with Tukey post hoc analysis. Non-parametric Kruskal–Wallis followed by Dunn’s post hoc test was used to measure data that violated the normal distribution assumption. The statistical analysis was carried out by using GraphPad Prism 7.00 for Windows. The data shown were presented as mean ± SEM with *p* < 0.05 regarded as the statistically significant difference at 95% confidence.

## 3. Results

### 3.1. Overview of Experimental Design and Instrument Setting

To increase the test throughput, we set up four transparent acrylic containers with 20 cm (L) × 20 cm (W) × 10 cm (H) dimension and equipped them with four top lights and one bottom light (LED plate) sources to provide constant light intensity (Figure 1A and Figure A1). A luminometer was used to measure the light intensity from either transmission light or reflection light (Figure 1B). The outlooking of fish images captured by using either conventional or infrared CCD is shown in Figure 1C. Seven experiments were conducted in order to understand the optimized color preference testing conditions (Figure 1D,E). For experiment 1, the potential light position effect on color preference was investigated to see whether the zebrafish exhibits distinct innate color preference when external light sources were provided from different positions. For experiment 2, the potential animal density effect was investigated to see whether the zebrafish exhibits distinct innate color preferences when they stay in different animal-to-space ratio conditions. For experiment 3, the potential social interaction (shoaling) effect was investigated by housing either single or six fishes in the same tank. For experiment 4, the potential gender effect was examined by conducting an innate color preference test using all male or female fishes. For experiment 5, the potential age effect was investigated by conducting a color preference test with zebrafish aged either 3-, 5- or 12-month-olds. For experiment 6, the potential strain effect was studied by conducting innate color preference tests with different strains of AB, TL, golden, absolute, and WIK. For experiment 7, the potential toxicological effect of ethanol on innate color preference was examined by exposing zebrafish (AB strain) to 1% ethanol for either 24 h or 96 h.

### 3.2. Experiment 1: Light Source Position on Zebrafish Color Preferences

**Figure 2:**
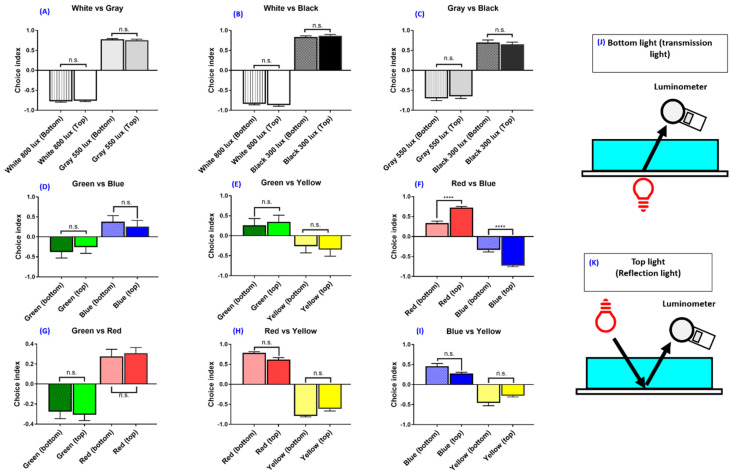
The effect of light intensity and light source position on the zebrafish swimming activity choice index. (**A**–**C**) The effect of different light intensity on the zebrafish swimming activity choice index. (**A**) White and grey combination, (**B**) white and black combination, (**C**) grey and black combination. (**D**–**I**) The effect of different color and light position on the zebrafish swimming activity choice index. (**D**) Green vs. blue combination, (**E**) green vs. yellow combination, (**F**) red vs. blue combination, (**G**) green vs. red combination, (**H**) red vs. yellow combination, (**I**) blue vs. yellow combination. (**J**,**K**) Schematic showing the light source positions either from the bottom or on top. The light intensity was measured by using a luminometer. The data are presented as mean ± SEM, *n* = 24 for each group. The difference was tested by one-way ANOVA and the significance level was set at, **** *p* < 0.0001. n.s. = non-significant.

Various conditions of the zebrafish innate color preference tests used in separate laboratories sometimes lead to contradictory results in published studies (Table A3). We hypothesized that the light source position in the color preference test might affect zebrafish color preferences. To test the hypothesis, first, we compared the achromatic color plate with different light positions to determine whether different light sources affect zebrafish color preference. We found that the light source did not result in a significant difference with positions in achromatic plate comparison. In a white (0.000691 uWatt/nm) and grey (0.000207 uWatt/nm) combination, the zebrafish preferred to stay in the grey partition (Figure 2A). The white–grey combination (*F* (3,92) = 1651, *p* = 0.9343) showed no significant difference in effect whether the light source was placed at the bottom or on top (Figure 2A). In a white–black (0.0000342 uWatt/nm) combination, the zebrafish preferred the black partition (Figure 2B). The light source position also showed no significant effect on color preference in the white–black combination (*F* (3,92) = 1249, *p* = 0.8649) as seen in Figure 2B. In a grey and black comparison, the zebrafish preferred the black partition (Figure 2C). No significant effect on color preference was detected for top and bottom light position (*F* (3,92) = 190.7, *p* = 0.9345) in grey–black color combination (Figure 2C). This result showed zebrafish preferred to stay in a lower lux intensity partition regardless of different plate combinations and light positions.

Based on the above findings, we tested whether the innate color preference in adult zebrafish has an associated light position effect. We addressed this question by using a color plate combination and performed an innate color preference test with either a top (Figure 2K) or bottom (Figure 2J) light source. We subjected all four-color plates on two color combinations in each tank, so the total color combinations were six color combinations. Each color combination was tested either with a top light source or a bottom light source. Results showed that the bottom and top light sources have similar color preference ranking patterns (from most to least: red > blue > green > yellow). The green–blue (*F* (3,92) = 145.1, *p* = 0.0292) (Figure 2D), green–yellow (*F* (3,92) = 105.9, *p* = 0.2851) (Figure 2E), green–red (Figure 2G), red–yellow (*F* (3,92) = 394.6, *p* = 0.0195) (Figure 2H) and blue–yellow combinations (*F* (3,92) = 64.57, *p* = 0.0897) (Figure 2I) showed no significant differences in regard to color preference, regardless of whether the light source was placed at the bottom or on top. Interestingly, although the red–blue combination showed no difference in color preference ranking, it showed significant differences in color preference index (*F* (3,92) = 254.5, *p* = 0.0398). That is, the light source on top has a higher color preference index in red compared with the light source placed at the bottom (Figure 2F). Together, our results demonstrated the innate color preference ranking in zebrafish is red > blue > green > yellow, and this ranking is not associated with the light source provided either from the bottom (transmission light) or the top (reflection light) positions. Based on these results, using the top and bottom light sources makes the results much more stable and repeatable.

### 3.3. Experiment 2: Tank Size (Animal Density) on Zebrafish Color Preferences

**Figure 3:**
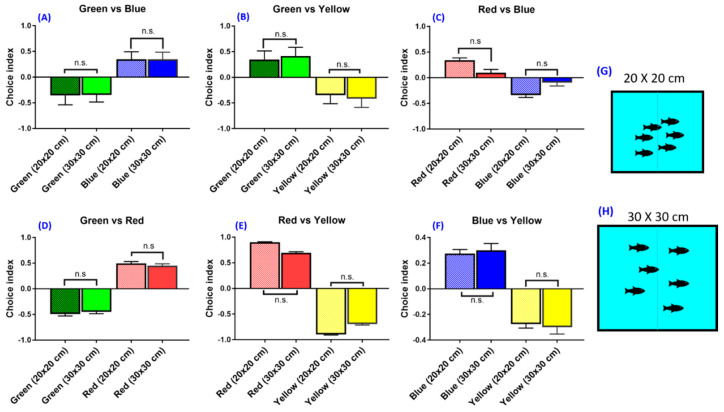
The effect of different tank sizes on the zebrafish swimming activity choice index. (**A**) Green vs. blue combination, (**B**) green vs. yellow combination, (**C**) red vs. blue combination, (**D**) green vs. red combination, (**E**) red vs. yellow combination, (**F**) blue vs. yellow combination. (**G**,**H**) Schematics showing two settings with different fish-to-tank ratios for assessment of the fish density effect. The data are presented as mean ± SEM, *n* = 24 for each group. The difference was tested by one-way ANOVA. n.s. = non-significant.

We also assessed whether the density of the tested animals plays a major role in color preferences which may cause the unrepeatability of the data. To reach this goal, zebrafishes were housed in different size containers at different fish-to-tank area ratios. The innate color preference for zebrafish housed in either a 20 × 20 × 10 cm tank (fish-to-tank ratio is 1:103, Figure 3G) or a 30 × 30 × 10 cm tank (fish-to-tank ratio is 1:224, Figure 3H) was measured and compared. Both the top light and bottom light sources were used simultaneously to get rid of any inconsistent factors in illumination. The fish-to-tank ratio was measured by using ImageJ software based on their relative pixel area. We found that there was no significant difference in either color preference ranking or index when using a 20 × 20 × 10 cm tank or a 30 × 30 × 10 cm tank in all color combinations tested (Figure 3A–F).

### 3.4. Experiment 3 and 4: Social Interaction and Gender on Zebrafish Color Preference

**Figure 4:**
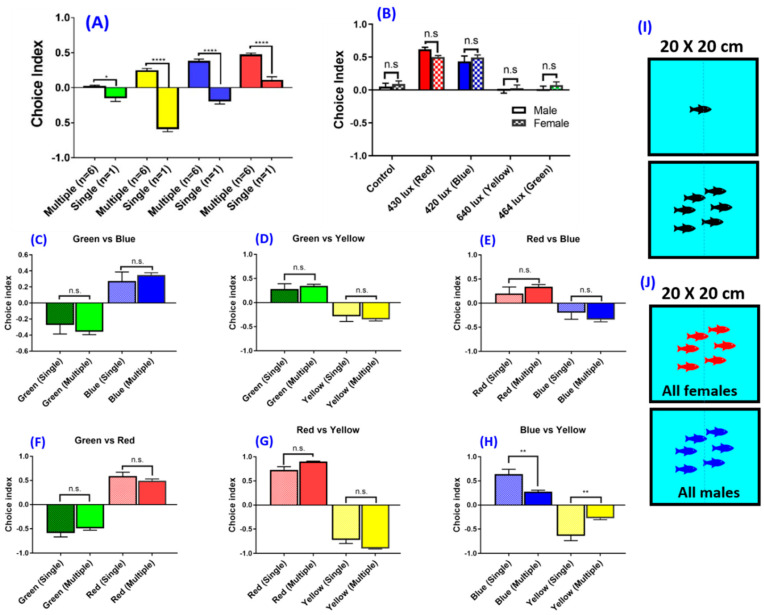
The effect of social interaction and gender on color preference using the top light source. (**A**) The effect of the single fish compared with multiple fish in a single tank on color preference using a blank-color partition. (**B**) The effect of gender on color preference using the light source on top. (**C**–**H**) The effect of social interaction on color preference using color combinations. (**C**) Green vs. blue combination, (**D**) green vs. yellow combination, (**E**) red vs. blue combination, (**F**) green vs. red combination, (**G**) red vs. yellow combination, (**H**) blue vs. yellow combination. (**I**) Schematics showing two settings in which either single or multiple fish were kept in a single tested tank. (**J**) Schematics showing two settings in which either six male or female fishes were kept in a single tested tank. The data are presented as mean ± SEM, *n* = 24 for each group. The difference was tested by one-way ANOVA and the significance level was set at * *p* < 0.05, ** *p* < 0.01, **** *p* < 0.0001. n.s. = non-significant.

In order to maximize the assay throughput, we measured the locomotor activity of six fishes in the same container (Figure 4I). With the aid of locomotion tracking software, it is possible to perform multiple fish tracking in a single arena to increase the experimental throughputs. When multiple zebrafish swim together, they will display shoaling behavior to reduce anxiety and lower the risk of being captured by predators [12,26,27]. However, zebrafish color preference experiments in previous studies were often conducted with a single fish in a color preference chamber or maze [2,3]. Whether multiple fish and single fish display different color preferences is an interesting and unanswered question. By analyzing multiple fish, with the aid of idTracker software for individual identity recognition, our results showed that the single fish and multiple fish tested in a single arena showed a significant difference in color preference (Figure 4A). In a tank experiment with blank-color partition, the single fish showed avoidance of green, yellow and blue. Blank partition means a transparent partition or without a color plate. Thus, we obtained four color combinations: blank–red, blank–blue, blank–green, and blank–yellow combinations. On the contrary, when the test was given to a group of fish, green, yellow, blue and red were preferred, which showed an opposite preference between the color partitions.

We also used a color plate combination to investigate whether the multiple fish and single fish display different color preference rankings. We used four color plates, namely, red, blue, green and yellow, with a total of six color combinations in this social interaction experiment. The result showed that there was no significant difference between single and multiple fish color preference ranking in color combination, but the variances in the single fish were higher compared with the multiple fish experiment conditions (Figure 4C–H). Together, our results showed that the color preference data obtained from a test conducted with multiple fish are more reproducible and consistent compared to those in which a single fish was tested.

To date, the potential gender effect on color preference in zebrafish has not been adequately addressed [11]. To this end, we used sex-maturated males and females aged 6–8 months to investigate the potential gender-associated color preference difference (Figure 4J). The results showed that there was no significant difference in color preference between male and female zebrafishes tested in this study. The innate color preference ranking and index showed no significant difference between genders (Figure 4B). Therefore, in the subsequent experiments, zebrafishes with mixed gender were used to conduct color preference experiments.

### 3.5. Experiment 5: Age Effect on Zebrafish Color Preferences

**Figure 5:**
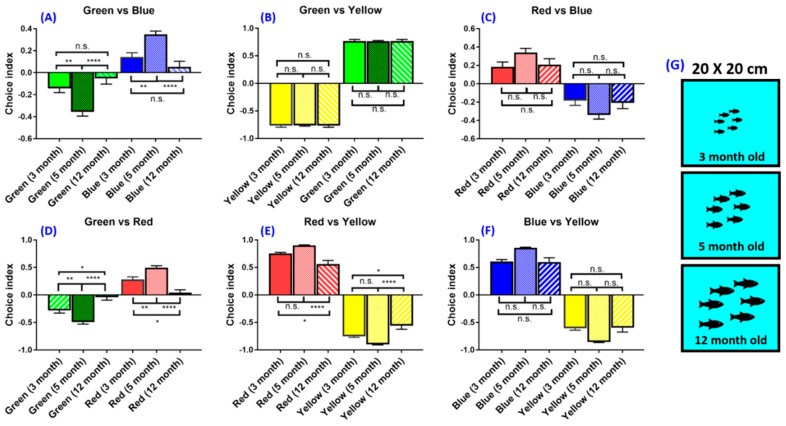
The effect of different ages on zebrafish color preference using the light source on top. (**A**) Green vs. blue combination, (**B**) green vs. yellow combination, (**C**) red vs. blue combination, (**D**) green vs. red combination, (**E**) red vs. yellow combination, (**F**) blue vs. yellow combination. (**G**) Schematics showing three settings with zebrafishes aged at either 3, 5 or 12 months old. The experiment was conducted with six zebrafishes inside one tank. The data are presented as mean ± SEM, *n* = 24 for each group. The difference was tested by one-way ANOVA and the significance level was set at * *p* < 0.05, ** *p* < 0.01, **** *p* < 0.0001. n.s. = non-significant.

A thorough review of the literature indicates that the potential effects of fish age on innate color preferences have not been carefully investigated in previous studies (Table A3). In this study, zebrafish with different ages from 3, 5 to 12 months old were subjected to color preference tests (Figure 5G). Results showed that their innate color preference ranking were the same (from most to least: red > blue > green > yellow) but the time they spent in the preferred color partition was different (Figure 5A–F). Notably, 12-month-old fish showed a relatively lower color preference index in blue–green (*F* (5,138) = 33.88, *p* = 0.4729) (Figure 5A) and red–green color combinations (*F* (5,138) = 61.39, *p* = 0.8010) (Figure 5D). However, the 3-month-old fish showed a relatively lower preference index compared with adult zebrafish in green–blue (*F* (5,138) = 33.88, *p* = 0.6579) (Figure 5A) and green–red (*F* (5,138) = 61.39, *p* = 0.0044) (Figure 5D) color combinations. It appears that the ability of the 3-month-old fish to discern color was higher compared with the 12-month-old fish because the time the 3-month-old fish chose their preferred colors was higher. There are also other possibilities to explain the differences that occurred in this experiment. Differences in the choice index may not be due to color preferences, but because of the fear or anxiety response. Old zebrafish could show fear or anxiety response to some colors and will likely show a freezing response and stay in the same color area. However, if the fish did not show a freezing response, then it could be interpreted as the color preference of the fish. In addition, the 5-month-old fish has the highest choice index in the green–blue combination (*F* (5,138) = 33.88, *p* = 0.0049) (Figure 5A) and green–red combination (*F* (5,138) = 61.39, *p* = 0.0163) (Figure 5D). Our result suggested that the optimal age for the color preference test in zebrafish is around 5 months old because they showed the highest choice index compared with other age groups.

### 3.6. Experiment 6: The Strain- and Species-Specific Effect on Zebrafish Color Preference

**Figure 6:**
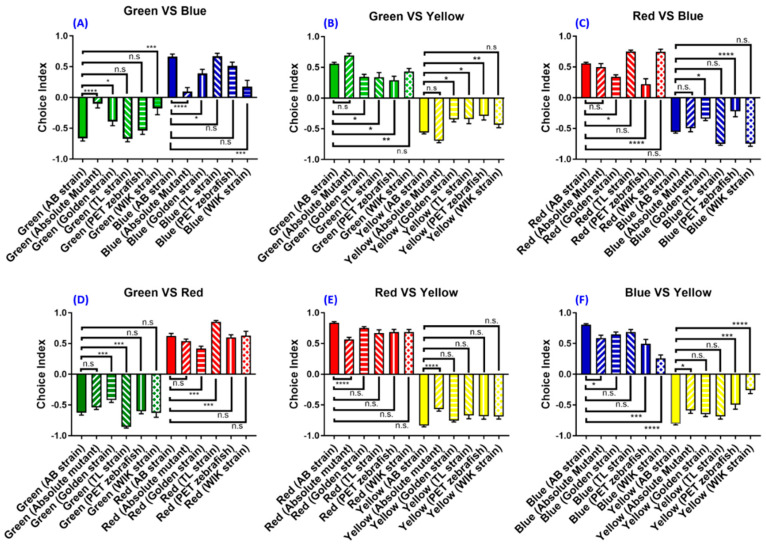
Color preference ranking and index difference between six different zebrafish strains. The color preference index for the (**A**) green vs. blue, (**B**) green vs. yellow, (**C**) red vs. blue, (**D**) green vs. red, (**E**) red vs. yellow, (**F**) and blue vs. yellow combinations. The data are presented as mean ± SEM, *n* = 24 for each strain, except for the Wild Indian Karyotype (WIK) strain (*n* = 12). The difference was tested by one-way ANOVA and the significance level was set at * *p* < 0.05, ** *p* < 0.01, *** *p* < 0.001, **** *p* < 0.0001. n.s. = non-significant.

The most popular strain that has been used for color preference tests in previous studies is the AB strain (Table A3). Some previous reports used zebrafish from the local pet store and the variation in the fish genetic background might contribute to the inconsistent color preference test results seen in the literature. Here, we tested the hypothesis by examining the innate color preference among six different zebrafish strains, namely, AB, absolute, golden, TL, PET, and WIK for the first time. Using the optimized condition established in this study, we discovered that all six zebrafish strains display the same color preference, with the ranking from most to least as red > blue > green > yellow. However, the color preference index for each strain displayed a significant difference compared to their AB strain counterpart (Figure 6A–F).

Compared to the AB strain, the absolute mutant exhibited a lower choice index in green–blue (*F* (11,252) = 296.7, *p* = 0.5117) (Figure 6A), red–yellow (*F* (11,252) = 374.1, *p* = 0.0181) (Figure 6E), and blue–yellow color combinations (*F* (11,252) = 189.3, *p* = 0.0457), respectively (Figure 6F). The reduction in the choice index in green–blue combination suggests that the absolute mutant spent less time in the blue color compartment and more time in green compartments. A similar reduction in color choice indices can also be found in red–yellow and blue–yellow combinations (Figure 6A, E, F). Compared to AB, the golden strain manifests different choice indices in green–yellow (*F* (11,252) = 84.61, *p* = 0.0273) (Figure 6B), red–blue (*F* (11,252) = 111.4, *p* = 0.0215) (Figure 6C), and green–red (*F* (11,252) = 296.7, *p* = 0.0009) (Figure 6D) color combinations. Compared to the AB strain, the TL strain showed a reduced choice index in the green–yellow color combination (*F* (11,252) = 84.61, *p* = 0.0208) (Figure 6B). They also exhibit a higher choice index in the green–red color combination (*F* (11,252) = 296.7, *p* = 0.0001), which means that the TL strain prefers red and spent most of their time in the red area compared to the green area (Figure 6D). Different wild-type breeds such as the PET strain showed identical color preference rankings with the AB strain. However, the PET strain exhibited a lower choice index in the green–yellow color combination test (*F* (11,252) = 84.61, *p* = 0.0020), indicating that the PET strain zebrafish spent more time in the yellow compartment compared to the AB strain (Figure 6B). Similar results also were seen in the red–blue color combination (*F* (11,252) = 111.4, *p* = 0.0001) (Figure 6C) and the blue–yellow color combination (*F* (11,252) = 189.3, *p* = 0.0002) for the PET strain (Figure 6F). Finally, the WIK strain exhibited the same color preference ranking with their AB strain counterpart, but with reduced choice indices in some color combinations such as green–blue (*F* (11,252) = 74.17, *p* = <0.0001) and blue–yellow (*F* (11,252) = 189.3, *p* = <0.0001) (Figure 6A,F). In conclusion, by using the established protocol and conditions, we provided solid evidence to show that the color preference in six tested zebrafish strains (AB, absolute, golden, TL, PET, and WIK) display different color preference indices, however, the major color preference ranking does not appear to be associated with their genetic backgrounds.

**Figure 7:**
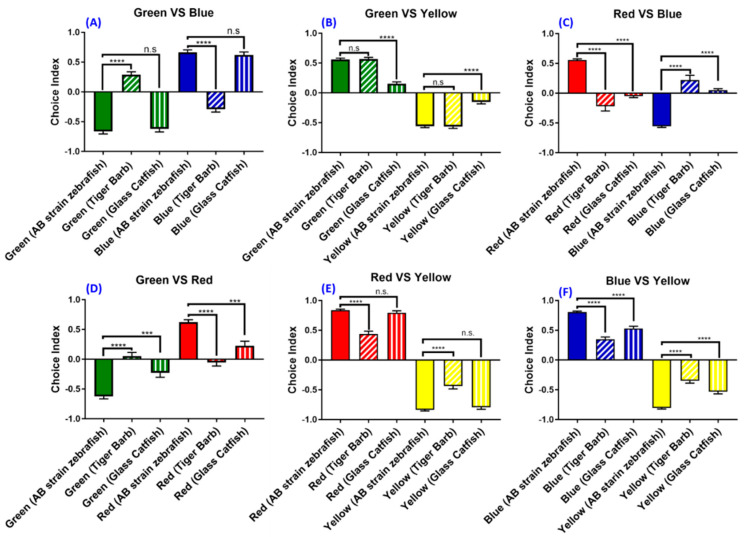
Color preference ranking and index difference for the tiger barb (*Puntigrus tetrazona*) and glass catfish (*Kryptopterus vitreolus*). The color preference index for the (**A**) green vs. blue, (**B**) green vs. yellow, (**C**) red vs. blue, (**D**) green vs. red, (**E**) red vs. yellow, (**F**) and blue vs. yellow combinations. The data are presented as mean ± SEM, *n* = 24 for each fish species. The difference was tested by one-way ANOVA and the significance level was set at *** *p* < 0.001, **** *p* < 0.0001. n.s. = non-significant.

Next, we asked whether the current optimized setting can be used to explore the color preference ranking and index for other small fish species. Two freshwater fishes, the tiger barb (*Puntigrus tetrazona*) and glass catfish (*Kryptopterus vitreolus*), with a similar body size to zebrafish were used in this study to explore the generality of the optimized experimental setting. Results showed that glass catfish display a similar color preference ranking with the AB strain zebrafish. It is intriguing to note that the tiger barb displays a distinct color preference ranking differing from that of the AB strain zebrafish. For the tiger barb, the innate color preference ranking is green > blue > red > yellow, while the AB strain zebrafish ranking is red >blue > green >yellow. The result in green–blue (*F* (5138) = 175.5, *p* = <0.0001), green–red (*F* (5138) = 49.2, *p* = <0.0001), and green–yellow color combinations (*F* (5138) = 358.3, *p* = <0.0001) indicate that green is the most preferred color for the tiger barb (Figure 7A,B,D). Similar to zebrafish, yellow is still the least preferred color for the tiger barb, which is corroborated by data collected from green–yellow, red–yellow, and blue–yellow color combinations (Figure 7B,E,F). In addition, the tiger barb exhibits lower choice indices in some color combinations, such as red–yellow (*F* (5138) = 429.1, *p* = <0.0001) and blue–yellow (*F* (5138) = 421.4, *p* = <0.0001) (Figure 7E,F).

For glass catfish, the innate color preference ranking is red >blue > green >yellow. The order is maintained as in zebrafish. This conclusion was supported by data collected from green–blue (*F* (5138) = 175.5, *p* = 0.9846), red–blue (*F* (5138) = 56.86, *p* = <0.0001), and blue–yellow color combinations (*F* (5138) = 421.4, *p* = <0.0001), where the blue choice index was higher than other colors (Figure 7A,C,F). Yellow is still the least favorite color of glass catfish, similar to the zebrafish and tiger barb (Figure 7). It can be seen that the yellow choice index in either green–yellow (*F* (5138) = 358.3, *p* = <0.0001), red–yellow (*F* (5138) = 429.1, *p* = 0.9604), or blue–yellow color combinations is the lowest compared to the other colors (Figure 7B,E,F). Together, our data clearly showed that the optimized experimental setting established in the present study can be applied to the innate color preference test conducted in other fish species.

### 3.7. Experiment 7: Effect of Ethanol on Zebrafish Color Preference

**Figure 8:**
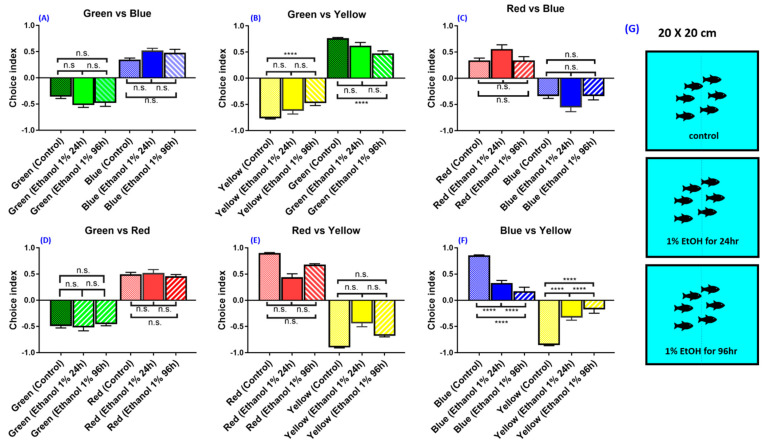
The effect of 1% ethanol on wild-type zebrafish with the light source on top. The ethanol at 1% concentration was systematically administered to zebrafish and their color preference changes were chronologically measured at 24 and 96 h. (**A**) Green vs. blue combination, (**B**) green vs. yellow combination, (**C**) red vs. blue combination, (**D**) green vs. red combination, (**E**) red vs. yellow combination, (**F**) blue vs. yellow combination. (**G**) Schematics showing three settings with either control or 1% ethanol exposure for 24 h or 96 h. The data are presented as mean ± SEM, control (*n* = 24); 1% ethanol, 24h (*n* = 12); 1% ethanol, 96h (*n* = 12). The difference was tested by one-way ANOVA and the significance level was set at **** *p* < 0.0001. n.s. = non-significant.

By using the optimized conditions described above, we aim to test the potential effect of environmental pollutants on color preference in zebrafish. In this experiment, we used ethanol as the potential pollutant to see their effects on color preference. Ethanol is extensively applied as a solvent in the industrial, research, and bioengineering process [28,29,30]. Excessive use of ethanol in industrial or research laboratories will likely become a new problem in the environment. Ethanol consumption has proved to have influence on the vestibulo-ocular system [31,32,33,34]. It also exhibits positional nystagmus and pursuit eye movements that further cause disturbed visual suppression [35,36,37,38]. Another experiment in zebrafish also proved that ethanol exposure to zebrafish embryos caused abnormalities of eye characteristics and affected the function of photoreceptors [39]. There is also a previous experiment that provides evidence that ethanol may affect visual systems, such as eye movements and fusion [40]. With all this evidence, we want to find out the effect of ethanol as an environmental pollutant on the color preference of zebrafish, as ethanol is known to affect the visual capabilities of animals and humans. Here, we took ethanol as an example to demonstrate chronological changes in the color preference of adult zebrafish after systematically being exposed to 1% ethanol. The AB strain zebrafish aged 5-month-old were exposed to 1% ethanol for either 24 h or 96 h. For most of the color combinations, the color preference ranking showed no significant change over time. This result suggests short-term ethanol exposure did not change the innate color preference ranking in zebrafish. However, it is intriguing to note that the color choice index in 1% ethanol-exposed zebrafish displayed a significant decrease in green–yellow (*F* (5,90) = 424.1, *p* = 0.0580) (Figure 8B) and blue–yellow combinations (*F* (5,90) = 307.6, *p* = <0.0001) over time (Figure 8F). In conclusion, the optimized protocol and settings for color preference ranking or index testing in zebrafish promises an applicable toxicity assessment for potential pollutants.

## 4. Discussion

Discrepancies in zebrafish color preference test results may have resulted from different factors that were not properly controlled and/or measured. The question of the potential influence of light intensity and light source position on color preference in zebrafish has not been adequately addressed. The zebrafish has been reported to prefer lower light intensity color, which is related to their strong preference towards blue [3]. Other parameters related to the living environment of the zebrafish may also affect color preferences.

In this study, carefully keeping the bottom light and top light at a similar light intensity, we found that the bottom light source and top light source gave similar results in both black–white and the color preference ranking and choice index (Figure 2). By careful control of the light position, intensity, and with a larger sample size, the zebrafish used in this study showed a preference for staying in the red and blue partitions, followed by staying in the green partition and avoiding the yellow partition. We concluded that the light intensity should be kept consistent in order to reduce bias for innate color preference in the zebrafish. However, the light source position did not affect zebrafish color preference.

Rhesus monkeys, chickens, turkeys, and mice have been reported to avoid red and yellow colors. These colors may be recognized as warning signals [41]. It has been reported that the color of feed may affect the behavior of zebrafish [12]. Avoidance of yellow discovered in the present study is in line with earlier studies showing that zebrafish tend to avoid yellow [1,7,14]. Yellow has a higher light intensity compared with the other colors, which may contribute to the avoidance behavior in the present study. Meanwhile, we found that red is the most preferred based on our zebrafish color preference test. Studies have shown that animals often prefer the color that is similar to or contrasts with the environment [42,43]. Animals tend to approach common colors that may help them find abundant food, while preference for a color that contrasts with the background may help animals to assess mate quality or to locate less common resources [44]. In addition, the preference towards red may be related to the color of the diet (red-colored pellet and artemia). Another study also showed that the zebrafish has an innate and relatively inflexible color preferences toward red [2].

We found that there is no significant effect of the tank size (animal density) used for the color preference test in the present study. This finding is in line with an experiment comparing differently sized tanks that had no significant effect in a novel tank test [45]. By using single fish in the single tank, we observed that the zebrafish had a different behavior compared to shoaling zebrafish. The single fish avoided going to color partitions even when the color partition had a lower light intensity compared to the blank partition, indicative of an altered behavior when the zebrafish were conditioned alone. A shoal of fish exhibited relaxed behavior when compared to the single fish scenario. We also observed that the freezing time for multiple fish was shorter as compared to that of a single fish. Such phenomenon may be affected by the social buffering effect of the zebrafish. Social interaction may ameliorate the anxiety level in zebrafish [46]. Animals’ ability to distinguish objects and colors in the environment is helpful in finding food and communicating in groups [47]. Our results showed that social interaction influences the color preference choice index in zebrafish behavior (Figure 4A) and is consistent with such interpretation. In the color combination experiment, although the color preference ranking of the zebrafish did not change, the data variation in the single fish experiment was higher compared with the multiple fish experiment (Figure 4C–H). Moreover, in the present study, we found that sex difference did not have a significant effect on color preference ranking, suggesting that conducting color preference experiments using the mixed gender should be considered as a valid condition (Figure 4B).

Age plays an important role in the color preference of zebrafish (Figure 5A–F). The 12-month-old fish were incapable of differentiating the green color partition when compared to the 3-month-old and 5-month-old fish. The 3-month-old and 5-month-old fish showed similar patterns of color preference ranking and choice index. It has been reported that the zebrafish is able to recognize visual stimuli from the early age of 5 dpf, highlighting the importance of visual perception [48]. Furthermore, the zebrafish was reported to have different light preferences between their larval stage and adult stage. The larval stage showed light preference, while the adult fish showed light avoidance [24,49,50]. The previous experiments in zebrafish also proved that visual acuity of the normal fish will be improved with age [51]. In the blue and green color combination tests, the 12-month-old zebrafish prefer the blue partition over green partition, which may be caused by aging. In other color combinations, the color preference index in older fish was lowered, which may be due to reduced color sensitivity. One of the known aging symptoms in the zebrafish is retina degeneration, which affects color perception [52]. Besides aging, another factor also could cause abnormal zebrafish visual behavior, such as environmental conditions like abnormal lighting environments [51]. Different preferences between larval and adult stages also can be caused by different opsin expressions between both stages. The zebrafish has two different opsin genes: The middle/long wavelength sensitive (M/LW) and *Rhodopsin-like* (*RH2*) opsin genes [53]. Both of them are expressed in different times and areas in zebrafish. Expression of LWS-2 (sensitive to a shorter wavelength) in juvenile and larval zebrafish is more expressed than LWS-1 (longer-wavelength sensitive) in the retina. Meanwhile, in adult zebrafish, LWS-2 expressions are lower than LWS-1, indicating a spectral shift of opsin type from short to long [53,54]. Differences in the larval and adult stages also can be caused by dissimilar photoreceptor development and function. Larval zebrafish only have cones as the functional photoreceptor in the retina until 10 dpf [55]. In the larval stage, a light-responsive cone photoreceptor does not appear until 4 days post-fertilization. Cones that are sensitive to color in zebrafish larva also do not mature until 10 days post-fertilization [56,57,58]. In our study, zebrafish prefer red and blue and avoid green and yellow, which is similar to previous studies [6]. However, there are differences in the choice index between young and old zebrafish. This could be the anxiety or fear behavior toward some color and not because old zebrafish could not recognize the color like young zebrafish. Freezing has been suggested as a measurement of anxiety [59]. A previous study proved that longer freezing was observed in zones in which fish spent more time in a specific color area. Time spent in each area demonstrated a tendency as a stay time of preferred colors. However, there are two possibilities of inducing freezing by color. It can be a tendency to avoid a specific color that reflects an anxiety or fear response. Another possibility is the behavior of the appetitive quality of another color, which is the behavior of desire to satisfy bodily needs and more preferred by zebrafish [59,60]. Older zebrafish may show an anxiety or fear response to some colors and stay in a specific color area. This tendency could be misinterpreted as a preferred color behavior. Nevertheless, it could be a tendency for appetitive quality. With this fact in mind, it is better to also test the freezing movement or freezing time in zebrafish caused by color in the future. Meanwhile, our result showed that the sexually matured 5-month-old fish were favorable to perform the color preference-related study on because of the established ability of distinctive specific color preference.

The most important finding of this study is that we were able to provide solid evidence to show that different zebrafish strains gave similar innate color preference ranking when compared with the AB strain, which has been used as the wild-type control in most experiments. However, each strain seems to exhibit a different choice index. Previous studies have shown that zebrafish color preferences could be different depending on their source population [61]. Fish evolution and development may be reflected in color preferences [62], suggesting that source population, developmental, and evolutionary history may play a role in fish color preference, as seen in the fact that each strain showed a specific choice index. Another study also suggests that evolved and developed zebrafish in different physical environments could have a different color preference, and these preferences could impact their learning abilities [61]. In another species, color may be an important factor in the prey choice of predators [63]. Different species will have different color preferences depending on their favorite prey or food. A certain study manages to examine the reaction of various fish species to different models of foods. They found out that each strain preferred a different color depending on their choice of foods [64]. Our results are consistent with these studies, where the tiger barb and glass catfish exhibit different color preferences compared to zebrafish. Each has a different preferred color. Specifically, the zebrafish prefers red, the glass catfish prefers blue and red, and the tiger barb prefers green. Some studies in cichlid fish showed that different colored environments could induce a variety of changes to the retina, morphologically and physiologically, in support of our study that each species from different environments tends to have a specific color preference. Another study in the barramundi (*Lates calcarifer*) showed that different colored light environments could change fish color preference and shift their visual system. In line with our color preference results that different fish species from diverse environments could exhibit dissimilar color preference ranking or indices, the study also highlighted the relationship between organisms and their visual environment and suggested that color preference could influence animal growth [65].

Depression and anxiety have been linked with color preference changes in humans, and these changes have already been used to determine mental state in physiological studies [66]. Ethanol-treated fish showed a reduction in the choice index in the blue–yellow combination, similar to zebrafish depression behavior reported previously [14]. We also found that ethanol-treated fish exhibited a reduction in the choice index in the green–yellow combination. Ethanol has been known to cause anxiety and depression with long-term usage [67]. In this study, we demonstrated the potential application of the color preference assay for chemical toxicology in a behavioral study.

## 5. Conclusions

We have optimized the color preference test conditions to highlight the importance of keeping the light intensity constant and the advantage of using multiple shoaling settings to maximize the experimental throughput. We observed no significant effect due to different light positions or tank sizes in this study. We also showed that there was no gender effect on color preference in zebrafish. In addition, our results also highlight that the utilization of the color preference setting reported herein is suitable for performing toxicity assessment by analyzing their color choice indices in different color combinations. Together, we conclude that color preference is a sensitive marker that can be used to identify alteration caused by a compound or environmental change. Result in zebrafish strain tests showed that color preference ranking remains the same, and only the choice indices differ. However, different species such as the tiger barb and glass catfish could exhibit different color preference rankings. The optimized conditions established herein are thus generally applicable to evaluate potential toxicological or pharmaceutical effects of chemicals. A large number of the sample can be easily obtained by using this reported method (*n* = 24 for single videotaping), and we believe that the methodology proposed in the present study provides a robust tool for phenotypic screening for isolation of mutants with color preference deficiency in the future.

## Appendix A

**Table A1:** Illumination condition of the top and bottom light sources.

Color	Bottom Light Intensity (Lux)	Top Light Intensity (Lux)
Red	440	430
Yellow	450	533
Green	430	497
Blue	410	424

**Table A2:** Wavelength (spectrum), reflectance, and irradiance (emission intensity) of all color plates.

Color	Wavelength Spectrum (nm) and Maximum Wavelength	Reflectance (%)	Irradiance (uWatt/nm)
Red	625–740 (625)	97	0.000489
Yellow	565–590 (572)	99	0.000587
Green	500–565 (541)	100	0.000376
Blue	450–485 (456)	98	0.000358
White	380–700 (561)	100	0.000691
Gray	400–700 (564)	96	0.000207
Black	500–700 (588)	80	0.0000342

**Table A3:** Comparison of the conditions of the zebrafish innate color preference tests used in various laboratories.

Illumination Direction	Tank Type	Experimental Design	Fish Gender	Fish Strain	Color Preferences Ranking	References
Top	T-Maze with colored plastic sleeves	Single fish	N.D.	AB	Red = Green > Yellow = Blue = No color	[11]
	Two-chambered PP (23 × 15 × 15 cm)	*n* = 12				
Bottom (LED plate)	Transparent acrylic plastic sheeting with a thickness of 3 mm. The dimensions of the tank 230 × 150 × 150 mm	Single fish	N.D.	AB	Blue > White > Red	[13]
		*n* = 18				
Top	T-Maze	Single Fish	Male and Female	AB	Blue > Purple > Green > Yellow = Orange > Blank	[3]
		*n* = 7–10				
Bottom	T-maze setup	*n* = 12	N.D.	AB	Blue > Red > Green > Yellow	[7]
	(Noldus IT (Information Technology))					
Bottom	Cross maze	Larvae	N.D.	Wild-type purchased from local pet stores	Blue > Red > Green	[6]
		*n* = 40				
Top	20 l glass aquaria	Group of 10 fish	N.D.	Zebrafish obtained from a commercial supplier	Red > Green > >White > Blue	[2]
(Colored Light)	(40 × 25 × 30 cm)					
Bottom	50 cm diameter transparent plastic tanks were divided into four laterals compartments of a similar size of 7 cm	*n* = 12	N.D.	Zebrafish obtained from a local fish farm	Blue = Green > Red = Yellow	[1]
Top	Cross-maze with sidearm covered in different colors	Single fish	N.D.	AB	Blue > Red = Green > Yellow	[14]
		*n* minimum = 8				
